# On Chancrous Folliculitis of the Vulva, or Follicular Soft Chancre

**Published:** 1880-08

**Authors:** 


### ON CHANCROUS FOLLICULITIS OF THE VULVA, OR FOLLICULAR SOFT CHANCRE.

A PAPER on the above subject, by MM. Gouguenheim and Bruneau, based on observations made at the Lourcine, was read before the Societe Medicale des Hopitaux. The following are the authors' conclusions: 1. Chancrous folliculitis, or follicular soft chancre, occupies most often the external surface of the labia majora. 2. It presents a special (*boutonneux*) character, which has led to its being frequently confounded with simple acute vulvar folliculitis. 3. The follicular condition may persist throughout the whole duration of the disease. 4. The follicular condition may disappear after some days, and the lesion assume the appearance of the ordinary chancre. 5. Simple chancres often coincides with follicular chancre. 6. Chancrous folliculitis often succeeds an ordinary chancre, but the reverse equally takes place. 7. It may exist alone, independently of any other ulceration. 8. Affection of the inguinal glands is rare. 9. Chancrous folliculitis evolves itself in the space of three or four weeks. 10. It is inoculable. 11. Inoculation, contrary to what happens almost always with the ordinary soft chancre, presents a period of incubation lasting from eight to twenty days. 12. The diagnosis of this disease, when it is not accompanied by the ordinary soft chancre, is impossible without inoculation. 13. The majority of observations of simple acute suppurative folliculitis, reproduced in books, without the criterion of inoculation, ought to be looked upon as cases of chancrous folliculitis. 14. The existence of simple acute suppurative vulvar folliculitis is then very hypothetical. 15. Secondary ulcerous folliculitis may only be a follicular soft chancre produced on syphilitic soil.—*Gazette Hebdomadaire de Medicine et de Chirurgie.*

